# Short-Finned Pilot Whale Strandings Associated with Pilot Whale Morbillivirus, Brazil

**DOI:** 10.3201/eid2901.221549

**Published:** 2023-01

**Authors:** Samira Costa-Silva, Carlos Sacristán, Rodrigo M. Soares, Vitor L. Carvalho, Pedro V. Castilho, Marta J. Cremer, Ana Carolina Ewbank, Arícia Duarte-Benvenuto, Thalita Faita, Pedro E. Navas-Suárez, Jenyffer V. Vieira, Letícia G. Pereira, Carolina F. Alves, Gabriela C. Souza, Giulia G. Lemos, Natália Silvestre-Perez, José L. Catão-Dias, Lara B. Keid

**Affiliations:** Universidade de São Paulo, São Paulo, Brazil (S. Costa-Silva, C. Sacristán, R.M. Soares, A.C. Ewbank, A. Duarte-Benvenuto, T. Faita, P.E. Navas-Suárez, N. Sivestre-Perez, J. Catão-Dias);; Centro de Investigación en Sanidad Animal (CISA-INIA), CSIC, Madrid, Spain (C. Sacristán);; Associação de Pesquisa e Preservação de Ecossistemas Aquáticos, Caucáia, Brazil (V.L. Carvalho, L.G. Pereira);; Universidade do Estado de Santa Catarina, Laguna, Brazil (P.V. Castilho, C.F. Alves, G.C. Souza);; Universidade da Região de Joinville, Sâo Francisco do Sul, Brazil (M.J. Cremer, J.V. Vieira, G.G. Lemos);; Universidade de São Paulo, Pirassununga, Brazil (L.B. Keid)

**Keywords:** morbillivirus, herpesvirus, viruses, cetacean, pilot whale, stranding, South America, Brazil, *Suggested citation for this article*: Costa-Silva S, Sacristán C, Soares RM, Carvalho VL, Castilho PV, Cremer MJ, et al. Short-finned pilot whale strandings associated with pilot whale morbillivirus, Brazil. Emerg Infect Dis. 2023 Jan [*date cited*]. https://doi.org/10.3201/eid2901.221549

## Abstract

Cetacean morbillivirus (CeMV) causes illness and death in cetaceans worldwide; the CeMV strains circulating in the Southern Hemisphere are poorly known. We detected a pilot whale CeMV strain in 3 short-finned pilot whales (*Globicephala macrorhynchus*) stranded in Brazil during July–October 2020. Our results confirm this virus circulates in this species.

Cetacean morbillivirus (CeMV; family Paramyxoviridae, genus *Morbillivirus*) is an important cause of illness and death in cetaceans (*1*). The genus *Morbillivirus* comprises 2 lineages: CeMV-1, which includes dolphin morbillivirus (DMV), porpoise morbillivirus (PMV), pilot whale morbillivirus (PWMV), and beaked whale morbillivirus (BWMV) strains; and CeMV-2, comprising the strain detected in Indo-Pacific bottlenose dolphins (*Tursiops aduncus*) in western Australia, the Fraser’s dolphin morbillivirus (FDMV), and Guiana dolphin morbillivirus (GDMV) strains (*1*,*2*). GDMV has been the only strain reported in cetaceans in Brazil (*3*). Four cases of PWMV have been recorded in pilot whales of the Northern Hemisphere, on the Atlantic coast of the United States and in the Canary Islands, Spain (*4*,*5*).

During July–October 2020, four short-finned pilot whales (*Globicephala macrorhynchus*) stranded in Brazil: 2 in Ceará state (cases 1 and 2) and 2 in Santa Catarina state (cases 3 and 4). All the animals stranded alive and died within 24 hours (Appendix Figure 1). We performed standard necropsies and collected tissue samples, which we fixed in 10% buffered formalin for histopathology or froze at −20°C or −80°C for molecular analysis.

We performed RNA extractions of all available tissues with TRIzol-LS (Life Technologies Corporation, https://www.thermofisher.com). We performed a morbillivirus 2-step reverse transcription nested PCR to amplify the phosphoprotein gene (*6*). After DNA extraction with the QIAGEN Blood & Tissue Kit (QIAGEN, https://www.qiagen.com), we performed herpesvirus detection in lung (n = 2) and liver (n = 4) samples by nested pan-PCRs to amplify DNA polymerase and glycoprotein B genes (*7*); when those were positive, we tested the remaining available tissues using the same protocols. We calculated percentage of identity among the obtained sequences and the closest ones from GenBank/EMBL/DDBJ based on p-distance. We used MEGA7 (https://www.megasoftware.net) to construct the phylogram (Figure).

**Figure:**
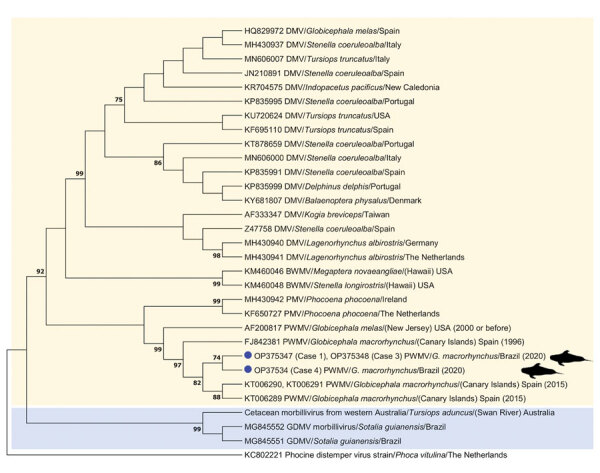
Maximum-likelihood phylogenetic tree based on Hasegawa-Khisino-Yano model with inversions gamma distribution and invariant sites of the phosphoprotein gene nucleotide sequences of cetacean morbillivirus PWMV obtained in Brazil (this study, blue circles), PWMV sequences previously described, and other morbillivirus strains described in cetaceans available from the GenBank/DDBJ/EMBL databases. Phocine distemper virus was selected as outgroup. The sequence identifier shows GenBank accession number, virus type, and location. Yellow shading indicates strains comprised in *Cetacean morbillivirus* lineage 1; blue shading indicates strains in lineage 2. Numbers at nodes indicate the bootstrap value; 1,000 bootstrap replications were selected, and bootstrap values <70 were omitted. BWMV, beaked whale morbillivirus; DMV, dolphin morbillivirus; GDMV, Guiana dolphin morbillivirus; PMV, porpoise morbillivirus; PWMV, pilot whale morbillivirus.

Three animals, cases 1, 3 and 4, were morbillivirus-positive, amplified in central nervous system, lung, and pulmonary lymph node samples (Table); sequences were submitted to GenBank (case 1, accession no. OP375347; case 3, OP375348; case 4, OP375349). Sequences from case 1 and 3 were identical and had a single nucleotide missense mutation (99.7% nt identity, 99.2% aa identity) when compared to the sequence from case 4. The sequences from cases 1 and 3 presented the highest nucleotide (99%) and amino acid identities (96.9%) with a PWMV sequence identified in 2 pilot whales in the Canary Islands, Spain (GenBank accession nos. KT006289 [animal 1], KT006290, and KT006291 [animal 2]). The sequence from case 4 had the highest nucleotide (99.2%) and amino acid similarities (97.7%) to the same PWMV sequences. Our sequences clustered with other PWMV sequences (Figure). In addition, we detected an alphaherpesvirus by the DNA polymerase protocol in a lung sample from case 3 (GenBank accession no. OP341880). The remaining tissue samples of case 3 (cerebellum, kidney, mesenteric lymph node, spleen, and liver) were herpesvirus-negative by PCR. The obtained herpesvirus has the highest similarity (99.5% nt identity, 100% aa identity) to an alphaherpesvirus obtained in a striped dolphin (*Stenella coeruleoalba*) from Spain (GenBank accession no. GQ888671). 

**Table:** Characteristics of PWMV-positive *Globicephala* pilot whales found in Brazil and other global locations*

Reference	Stranding date	Location	Body length, m	Age class/ sex	Gross pathology findings	Microscopic findings	PCR testing
Taubenberger et al. (*8*)	NA	Delaware and New Jersey, USA	NA	NI/F	Cachexia, dark red and congested lungs, parasites in stomach	Type 2 pneumocyte hyperplasia syncytial cells and some bronchiolar epithelial cells contained eosinophilic intracytoplasmic and intranuclear inclusion bodies	**Brain,** lung
Bellière et al. (*4*)	1996 Jul 28	Tenerife, Canary Islands, Spain	NA	Ju/M	Purulent pneumonia	Severe, diffuse, nonpurulent encephalomyelitis	**Brain,** urinary bladder
Sierra et al. (*5*)	2014 Jan	Fuerteventura, Canary Islands, Spain	2.02	Ju/F	Suppurative rhinitis, with clogged nasal passages by the accumulation of large quantity of purulent material, otitis media, sacculitis, and laryngitis. Severe diffuse epithelial hyperplasia and hyperkeratosis was observed along the upper respiratory tract and keratinized stomach	Dermatitis, bronchointerstitial pneumonia, laryngitis, laryngeal tonsillitis, hyperkeratotic gastritis, lymphoid depletion,	**Lung, larynx, pulmonary lymph node, laryngeal tonsil, spleen, intestine**
Sierra et al. (*5*)	2015 May	Fuerteventura, Canary Islands, Spain	1.68	Calf/M	Severe proliferative dermatitis and cheilitis, and severe, suppurative, laryngeal tonsillitis	Dermatitis, cheilitis, suppurative laryngeal tonsillitis, bronchointerstitial pneumonia, nonsuppurative meningoencephalitis with neuronal and glial cell degeneration and necrosis, microgliosis and syncytial cells	**Brain, laryngeal tonsil, lung, pulmonary lymph node, intestine**
This study, case 1	2020 Jul 13	Beberibe, Ceará state, Brazil	2.83	Ju/F	Cachexia, suppurative sinusitis, meningeal thickening, parasites in pterygoids sinus and pneumonia	Light multifocal Meningitis nonsuppurative, diffuse moderate neuronal and glial cell degeneration, cerebral edema, multifocal light bronchopneumonia and multiorgan passive congestion	Cerebrum, cerebellum, **spinal cord, lung**, heart, spleen, liver, pulmonary lymph node, mesenteric lymph node, pre-scapular lymph node, skin, kidney
This study, case 3	2020 Oct 4	Laguna, Santa Catarina state, Brazil	2.89	Ju/M	Poor body condition, dermatitis, and focal jaw fracture	Segmental hyaline skeletal myodegeneration and myonecrosis multifocal, light, acute tubular degeneration, adrenal gland with focal cortical hyperplasia, nonsuppurative colitis with moderated focal lymphoid hyperplasia and multiorgan congestion, astrocytosis and glial cell degeneration	Cerebrum, **cerebellum**, **spinal cord**, **liquor**, pre-scapular lymph node, tongue, trachea, lung, heart, spleen, liver, stomach, pancreas, lymph node mediastinal, lymph node mesentery, adrenal gland, kidney, gall bladder, skin
This study, case 4	2020 Oct 9	São Francisco do Sul, Santa Catarina state, Brazil	2.58	Ju/M	Poor body condition, tracheal edema and pneumonia	Multifocal light bronchointerstitial pneumonia with hyperplasia of pneumocytes type II, light diffuse nonsuppurative colitis, light diffuse lymphoid hyperplasia in thymus and spleen, light diffuse enteritis and colitis with moderated diffuse lymphoid hyperplasia and multiorgan congestion	**Brain,** heart, kidney lung, thymus, liver, **pulmonary lymph node**, muscles

*Bold text indicates PWMV-positive samples. Ju, juvenile; NA, not available; NI, not identified; PWMV, pilot whale morbillivirus.

The general health of the CeMV-positive animals was poor, and all were undernourished. We compared the main pathologic findings in these animals to all other cases of PWMV strain reported in the literature (Table).

Pilot whales are susceptible to DMV and PWMV; DMV caused atypical pilot whale deaths in the Mediterranean Sea (*6*). By contrast, 4 cases of PWMV infections have been recorded; 1 in New Jersey, USA, and 3 in the Canary Islands, Spain (*4*–*6*,*9*,*10*). All of them had multiorgan infections (*4*,*5*). Case 1 likely had a subacute or systemic CeMV infection characterized by meningomyelitis with gliosis and lymphocytic bronchointerstitial pneumonia. Further studies are necessary to elucidate if cases 3 and 4 manifested an infection similar to the brain-only DMV form or a systemic infection with heterogenic dissemination. The poor nutritional condition observed in all PWMV-positive animals could be the result of decreased foraging capacity caused by encephalitis (*1*). Case 3 had alphaherpesvirus and CeMV co-infection, a comorbidity previously reported in cetaceans, including pilot whales (*5*,*10*); in this case, however, there were no associated herpesviral lesions. All PWMV-positive cetaceans we described were juveniles, which could be associated with maternal passive immunity loss.

The occurrence of pilot whale strandings in 2020 on the coast of Brazil could be considered atypical. Of interest, although case 1 was stranded >3,300 km away from case 3 along the coastline, it had the same PWMV sequence type, which suggests circulation of that type along the coast of Brazil. Further studies are necessary to understand the effects and epidemiology of morbillivirus in cetaceans in the South Atlantic Ocean. However, the high similarity between our sequences and the PWMV detected in the Northern Hemisphere confirms that this strain also circulates in South America pilot whales and might be enzootic in *Globicephala* sp. whales in the Atlantic Ocean. 

Appendix Additional information from study of short-finned pilot whale strandings associated with pilot whale morbillivirus, Brazil.
