# Supplementary material for: Short-Finned Pilot Whale Strandings Associated with Pilot Whale Morbillivirus, Brazil
Source: Emerg Infect Dis. 2023 Jan;29(1):214–7. doi: 10.3201/eid2901.221549 (PMC9796215; doi:10.3201/eid2901.221549)
Supplement: Appendix — Additional information from study of short-finned pilot whale strandings associated with pilot whale morbillivirus, Brazil. [file 22-1549-Techapp-s1.pdf]

Article DOI: https://doi.org/10.3201/eid2901.221549

Short-Finned Pilot Whale Strandings Associated with Pilot Whale Morbillivirus, Brazil

Appendix

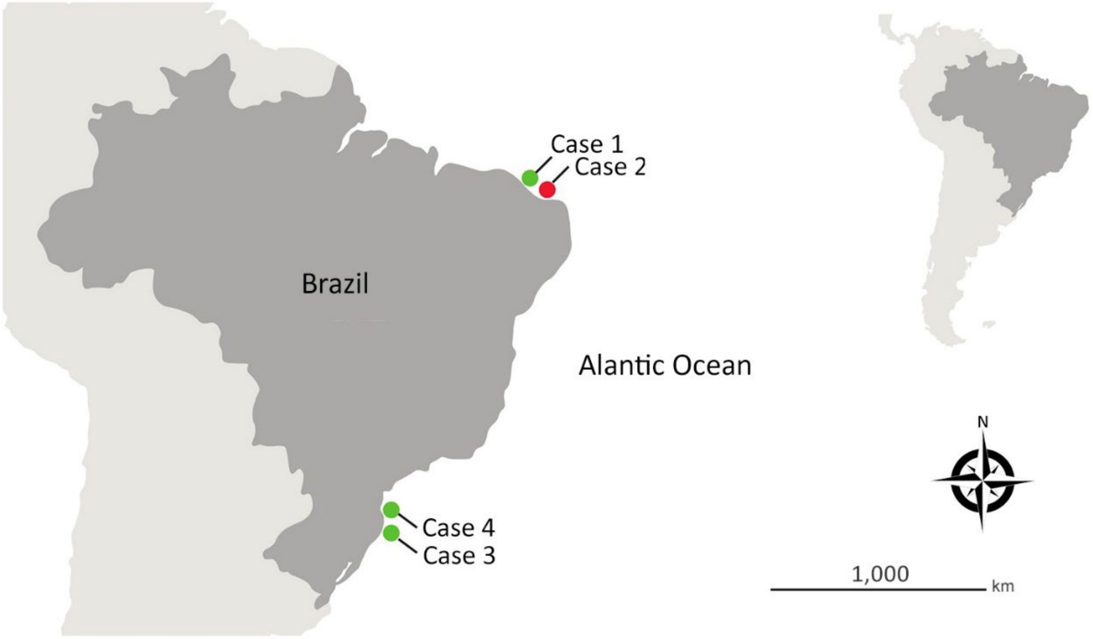

Appendix Figure. Short-finned pilot whale strandings along the Brazilian coast in 2020. Red dot represents the neonate pilot whale that tested negative for cetacean morbillivirus (case 2). Green dots represent the animals that tested positive for pilot whale morbillivirus (cases 1, 3, and 4).
